# Long non-coding ribonucleic acid H19 and ten-eleven translocation enzyme 1 messenger RNA expression levels in uterine fibroids may predict their postoperative recurrence

**DOI:** 10.6061/clinics/2021/e2671

**Published:** 2021-09-28

**Authors:** Xiangjuan Zhan, Hui Zhou, Yuhong Sun, Baomei Shen, Di Chou

**Affiliations:** I Department of Gynecology, The Second People's Hospital of Rizhao, Rizhao, Shandong, China.; II Department of Obstetrics and Gynecology, The Second People's Hospital of Dongying, Dongying, Shandong, China.; III Department of Oncology, Binzhou Hospital of Traditional Chinese Medicine, Binzhou, Shandong, China.; IV Department of Obstetrics and Gynecology, The People's Hospital of Pingyi County, Linyi, Shandong, China.

**Keywords:** Diagnostic Value, lncRNA H19, Postoperative Recurrence, TET1, UFs

## Abstract

**OBJECTIVES:**

To investigate the predictive value of long non-coding RNA (lncRNA) H19 and the ten-eleven translocation enzyme 1 (*TET1*) transcriptional expression in postoperative recurrence of uterine fibroids (UFs).

**METHODS:**

Seventy-five patients with UF, who underwent surgical treatment, were enrolled in the treatment group, and 60 healthy individuals were enrolled in the control group. The relative expression levels of lncRNA H19 and *TET1* mRNA in the serum and UF tissues were analyzed. The patients were further divided into a better curative (BC) group and a poor efficacy (PE) group to analyze the predictive value of lncRNA H19 and *TET1* and the independent risk factors affecting the recurrence of UF.

**RESULTS:**

Compared with the control group, lncRNA H19 expression levels were significantly higher, while *TET1* expression levels were significantly lower in the treatment group (*p*<0.001). The area under the receiver operating characteristic (ROC) curve (AUC) values of the two indicators for diagnostic importance were found to be 0.872 and 0.826, respectively. Compared with the PE group, lncRNA H19 expression levels were significantly lower, while *TET1* expression levels were significantly higher in the BC group (*p*<0.001). The AUC values of the two indicators for their predictive efficacy were 0.788 and 0.812, respectively. Logistic regression analysis showed that age, menarche age, maximum diameter of UFs, number of UFs, lncRNA H19 levels, and *TET1* levels were independent risk factors affecting UF recurrence. The AUC values of lncRNA H19 and *TET1* for their predictive value for postoperative recurrence were 0.814 and 0.765, respectively.

**CONCLUSIONS:**

The lncRNA H19 and *TET1* have high diagnostic and predictive efficacy for determining the postoperative recurrence of UFs.

## INTRODUCTION

Uterine fibroids (UFs) are benign uterine smooth muscle tumors that affect the fertility of women of childbearing age. The incidence of UFs varies with age, with 40-80% of women of childbearing age being diagnosed with this disease (1). Patients with UF may be asymptomatic or show severe and chronic symptoms, of which the most common ones include heavy menstrual bleeding, fatigue, pelvic pain, stress, back pain, frequent urination, constipation, and infertility (2,3). As many as 70% of white women and more than 80% of women of African descent are diagnosed with UFs in their lifetime, which has had a great impact on the global medical services and costs (4).

Surgical resection of UFs preserves fertility, so that patients can still be pregnant and deliver after surgery, which makes it a common therapeutic method (5). However, the recurrence rate of UFs has increased over the years following both open surgery and laparoscopic resection (6). There have been very few studies on the biological predictors of postoperative recurrence. Therefore, identifying biological indicators with a higher predictive value is critical for choosing optimal treatments and reducing the recurrence rate. Recent studies have revealed that the long non-coding RNA (lncRNA) H19 and ten-eleven translocation enzyme 1 (*TET1*) play important roles in the pathogenesis of UFs. LncRNAs are non-coding RNAs that are longer than 200 nt and are transcribed from DNA but are not translated into proteins; they regulate the expression of target genes (7) and play a pivotal role in the pathogenesis of epithelial ovarian cancer (8), osteosarcoma (9), and UFs (10). TET1 belongs to the demethylase family of proteins in mammals and hydrolyzes 5-methylcytosine (5-mC) into 5-hydroxymethyluracil (5-hmC) (11,12). Thus, it regulates gene expression by modifying histone marks and chromatin accessibility (13). Moreover, TET1 inhibits cancer progression (14), and its low expression has been associated with the invasion and metastasis of many malignant tumors (15-17). As lncRNA H19 and TET1 are both important regulatory factors in UFs (18), with lncRNA H19 expression acting as an estrogen receptor (ER) modulator and the plasma H19 levels being significantly correlated with the ER and progesterone receptor (PR) (19,20), we hypothesized that they are involved in the pathogenesis and postoperative recurrence of the disease.

However, little is known about the mechanism underlying the postoperative recurrence of UFs and the predictive value of potential indicators. In this study, we analyzed the circulating levels of lncRNA H19 and *TET1* mRNA in UFs and their predictive value for the postoperative recurrence of UFs. Our study provides an important reference for the clinical treatment of UFs in the future.

## MATERIALS AND METHODS

### Collection of clinical data

Seventy-five patients with UF who underwent surgical treatment in The People’s Hospital of Pingyi County, Linyi City, from February 2015 to March 2017 were enrolled in the treatment group, while 60 healthy individuals who underwent physical examination in our hospital during the same period were enrolled in the control group. The healthy controls were >18 years of age and had normal laboratory parameters without congenital immune deficiency.

### Inclusion and exclusion criteria

The inclusion criteria were patients diagnosed with UFs by imaging, based on the diagnostic guidelines (21), patients who met the indications for surgical resection, patients with complete clinical data, those who cooperated during the follow-up conducted in our hospital, and the patients and families who signed informed consent forms for participation in this study. This study was approved by the Medical Ethics Committee of the People's Hospital of Pingyi County.

Exclusion criteria: Patients with a previous history of pelvic surgery, malignant tumors, communication disorders, or hepatic and renal dysfunction; pregnant women; and patients who withdrew from the study before its completion.

### Sample collection and detection

Fasting venous blood (3 mL) was collected from the research subjects on the second day after enrollment. The blood was allowed to stand at room temperature for 30 min and then centrifuged (3000 g at 4°C for 10 min) to generate the supernatant, which was stored at −80°C for cryopreservation. UF tissues were collected from the patients during the operation, and all tissues were frozen in liquid nitrogen until further testing.

**Figure S1:**
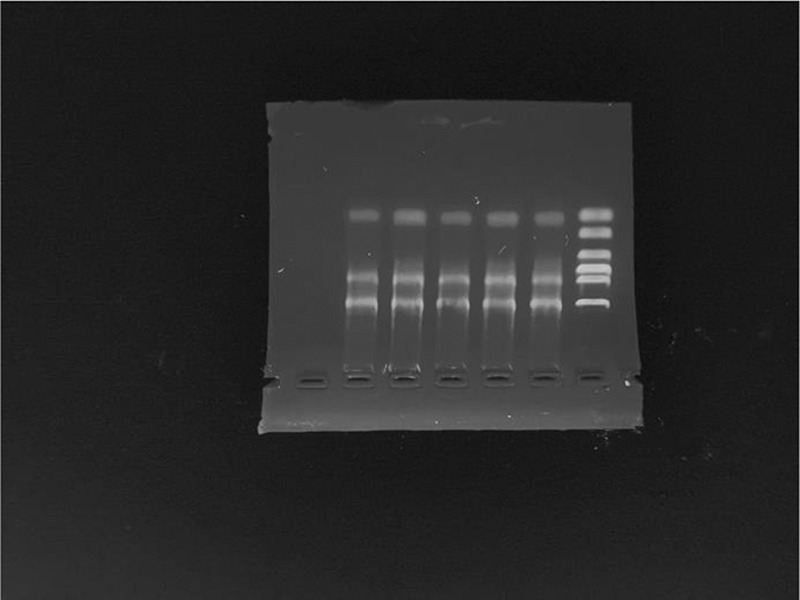
Gels showing the purity and integrity of the RNA samples.

TRIzol reagent (15596018; Invitrogen, Carlsbad, California, USA) was used to extract the total RNA from tumor-free RNA in the serum and tissues. UV spectrophotometry and agarose gel electrophoresis were used to confirm the purity, concentration, and integrity of RNA (Figure S1). Subsequently, reverse transcription was performed using the TaqMan Reverse Transcription Kit (N8080234; Invitrogen, Carlsbad, California, USA) with a reaction volume of 15 µL. Thermal cycling conditions were 16°C for 30 min, 42°C for 30 min, and 85°C for 5 min, followed by a 4°C hold. TransStart Green quantitative polymerase chain reaction (qPCR) SuperMix UDG (AQ111-01; Beijing TransGen Biotech Co., Ltd., China) was used to reverse transcribe the extracted total RNA, according to the manufacturer’s instructions. cDNA was collected for PCR amplification. qPCR reaction included cDNA (1 µL), upstream and downstream primers (0.4 µL each), 2× TransStart^®^ Green qPCR SuperMix UDG (10 μL), Passive Reference Dye (50×) (optional) (0.4 μL), and nuclease-free water that was finally added to a volume of 20 μL (Table 1). Amplification conditions involved incubation (94°C for 10 min), pre-denaturation (94°C for 5s), and annealing and extension (60°C for 30s), for a total of 40 cycles. cDNA from each sample was amplified in triplicate in three independent wells. U6 and glyceraldehyde 3-phosphate dehydrogenase (*GAPDH*) were used as internal references. The 2^-△△Ct^ method was used to calculate the relative gene expression levels.

### Outcome analysis

#### Main outcome analysis

Patients with UF were followed up for 2 years by telephone and outpatient visits to evaluate their postoperative recurrence, and the 2-year postoperative recurrence rate was analyzed (22). Binary logistic regression was conducted to analyze the independent risk factors that affect UF recurrence and the predictive value of the expression levels of lncRNA H19 and *TET1* for recurrence.

#### Secondary outcome analysis

The expression levels of serum lncRNA H19 and *TET1* were observed in the two groups. Receiver operating characteristic (ROC) curves were plotted to analyze the diagnostic value of the two indicators in the UFs. The short-term clinical efficacy of the operation was evaluated, and the efficacy evaluation criteria are listed in Table 2. The expression levels of lncRNA H19 and *TET1* in the UF tissues of patients in the better curative (BC) and poor efficacy (PE) groups were observed. The ROC curves were plotted to analyze the predictive efficacy of the two indicators in UFs.

### Statistical analysis

In this study, SPSS v.20.00 was used for statistical analysis, and GraphPad Prism 7.05 (https://www.graphpad.com/support/) was used for figure plotting. Data conforming to a normal distribution are expressed as the means±standard deviations (SDs). The comparison of lncRNA H19 and *TET1* levels in the serum between the operation and control groups, and the comparison of the levels in the UF tissues between the BC and PE groups were analyzed by t-test and represented by the t value. Categorical variables were expressed as percentages (%), analyzed using the chi-square test, and represented as χ^2^ values. With recurrence as a dependent variable and the factors with differences in the univariate analysis as independent variables, binary logistic regression was performed using the enter method to analyze the risk factors for UF recurrence. ROC curves were plotted to analyze the diagnostic and predictive efficacy values of lncRNA H19 and *TET1* in UFs for recurrence.

## RESULTS

### Comparison of general clinical data

There were no significant differences between the operation and control groups in age, body mass index (BMI), history of smoking, history of alcoholism, family history, or place of residence (*p*>0.05), indicating comparability of clinical data from the two groups (Table 3).

### Expression levels of lncRNA H19 and *TET1* mRNA in serum before operation

**Figure 1:**
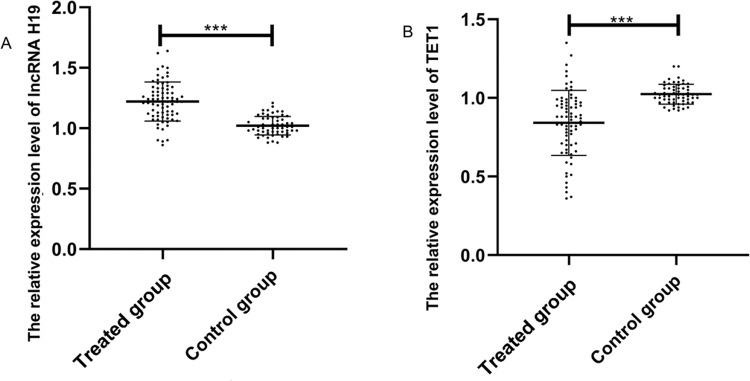
Relative expression levels of the long non-coding RNA (lncRNA) H19 and ten-eleven translocation enzyme 1 (*TET1*) in serum before operation. A. The relative expression levels of lncRNA H19 in the treated group were significantly higher than those in the control group. B. The relative expression levels of TET1 in the treated group were significantly lower than those in the control group. ***indicates *p*<0.001.

The expression levels of lncRNA H19 and *TET1* in serum were examined before the operation. The expression level of lncRNA H19 in the treated group was significantly higher than that in the control group (1.257±0.165 *versus* 1.025±0.078) (*p*<0.001), whereas *TET1* expression levels in the treated group were significantly lower than those in the control group (0.816±0.211 *versus* 1.022±0.058) (*p*<0.001) (Figure 1).

### Diagnostic value of lncRNA H19 and *TET1* in UFs

**Figure 2:**
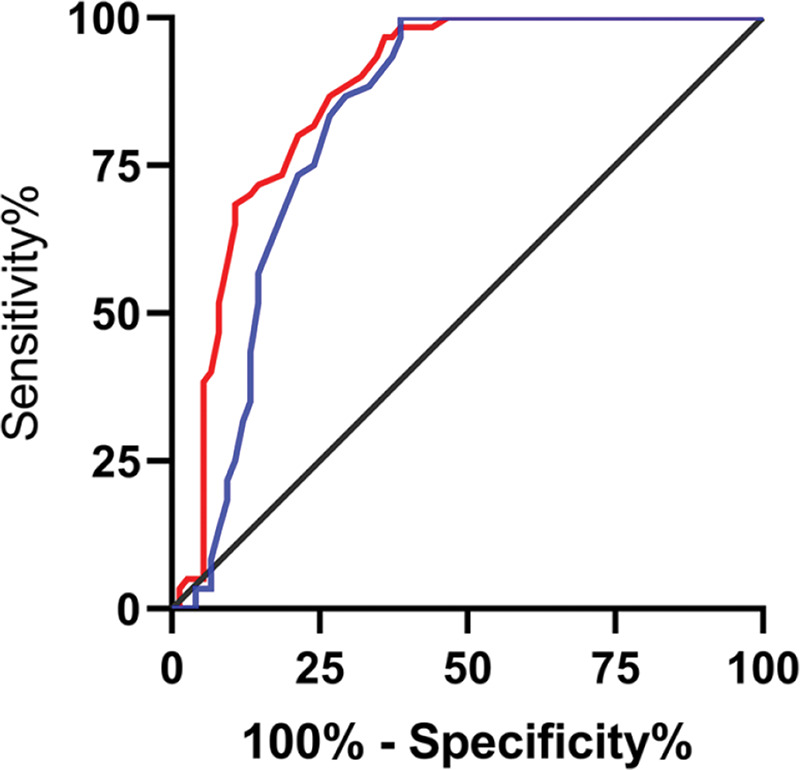
Diagnostic value of lncRNA H19 and *TET1* for uterine fibroids (UFs). A. The red line indicates the receiver operating characteristic (ROC) curve of the diagnostic value of lncRNA H19 in UFs, with the area under the curve (AUC), sensitivity, specificity, and cut-off value of 0.872 (95% CI: 0.811-0.933), 96.67%, 64.00%, and <1.160, respectively. B. The blue line indicates the ROC curve of the diagnostic value of *TET1* in UFs, with the AUC, sensitivity, specificity, and cut-off value of 0.826 (95% CI: 0.753-0.899), 100%, 61.33%, and >0.915, respectively.

The relative expression levels of lncRNA H19 and *TET1* in the serum before the operation were used to plot the ROC curves. The area under the curve (AUC) was 0.872 (95% CI: 0.811-0.933), with 96.67% sensitivity and 64.00%, and a cut-off value of <1.160. The AUC of *TET1* was 0.826 (95% CI: 0.753-0.899), with 100% sensitivity and 61.33% specificity, and a cut-off value of >0.915 (Figure 2).

### Predictive efficacy values of lncRNA H19 and *TET1* in UFs

**Figure 3:**
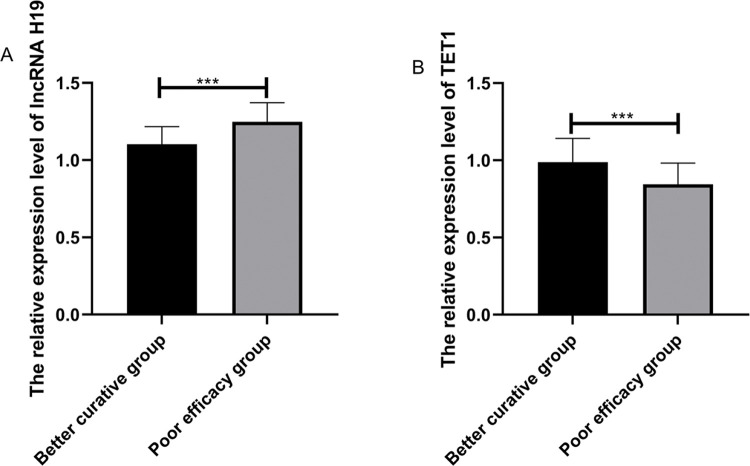
Relative expression levels of lncRNA H19 and *TET1* in UF tissues before operation. A. The relative expression levels of lncRNA H19 in the better curative (BC) group were significantly lower than those in the poor efficacy (PE) group. B. The relative expression levels of *TET1* in the BC group were significantly higher than those in the PE group. ***indicates *p*<0.001

**Figure 4:**
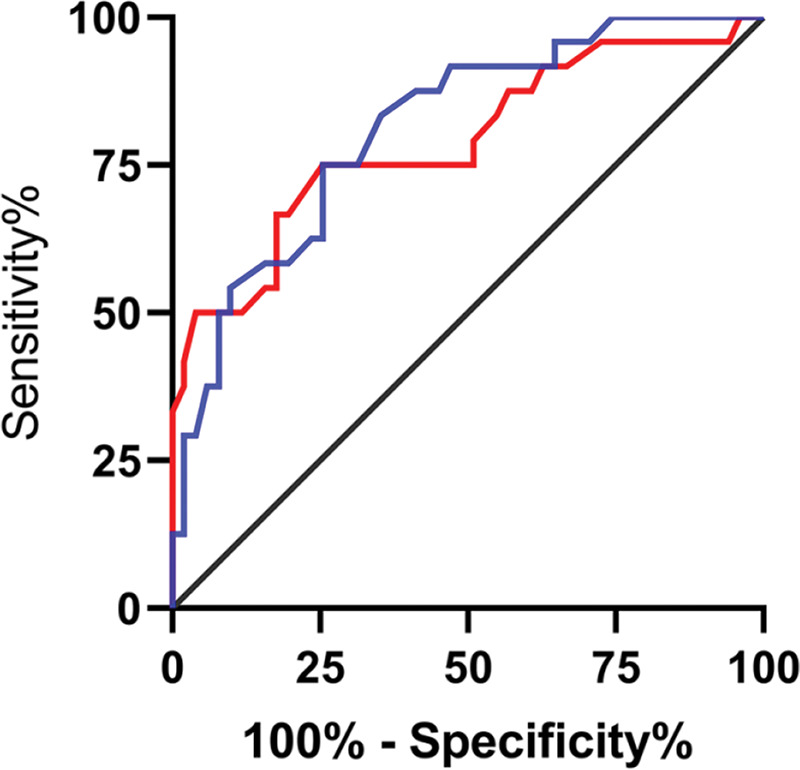
Predictive efficacy values of lncRNA H19 and TET1 for UFs. A. The red line indicates the ROC curve of the predictive efficacy value of lncRNA H19 in UFs, with an AUC, sensitivity, specificity, and cut-off value of 0.788 (95% CI: 0.669-0.907), 75%, 74.51%, and >1.155, respectively. B. The blue line indicates the ROC curve of the predictive efficacy value of *TET1* in UFs, with an AUC, sensitivity, specificity, and cut-off value of 0.812 (95% CI: 0.711-0.911), 75%, 74.51%, and <0.905, respectively.

Among the patients in the treated group, 51 (68%) were markedly effective, 18 (24%) were effective, and 6 (8%) were ineffective. The patients were divided according to the short-term clinical efficacy of surgery into the BC group (n = markedly effective) and the PE group (n=effective+ineffective). The expression levels of lncRNA H19 and *TET1* in UF tissues before surgery were analyzed. LncRNA H19 expression levels in the BC group were significantly lower than those in the PE group (1.103±0.114 *versus* 1.248±0.124) (*p*<0.001), whereas *TET1* expression levels were significantly higher than those in the PE group (0.988±0.154 *versus* 0.844±0.138) (*p*<0.001) (Figure 3). The expression levels of lncRNA H19 and *TET1* in the UF tissues before surgery were used to plot the ROC curves. The AUC of lncRNA H19 was 0.788 (95% CI: 0.669-0.907), and the AUC of *TET1* was 0.812 (95 % CI: 0.711-0.911) (Figure 4).

### Risk factors for postoperative recurrence

The 2-year postoperative recurrence of UFs was followed up in 75 patients, and based on the results, 22 patients (29.33%) were included in the disease recurrence (DR) group, while 53 (70.67%) were included in the no recurrence (NR) group. Univariate analysis of clinical data showed significant differences between the two groups in age, menarche age, maximum diameter of UFs, number of UFs, lncRNA H19 levels, and *TET1* levels (*p*<0.05) (Table 4). Next, factors with differences were assigned (Table 5). With recurrence as a dependent variable and risk factors as independent variables, binary multivariate logistic regression was conducted using the enter method to analyze independent risk factors for UF recurrence. The results showed that age, menarche age, maximum diameter of UFs, number of UFs, lncRNA H19 levels, and *TET1* levels were independent risk factors affecting UF recurrence (Table 6).

### Predictive values of lncRNA H19 and *TET1* for the postoperative recurrence of UFs

**Figure 5:**
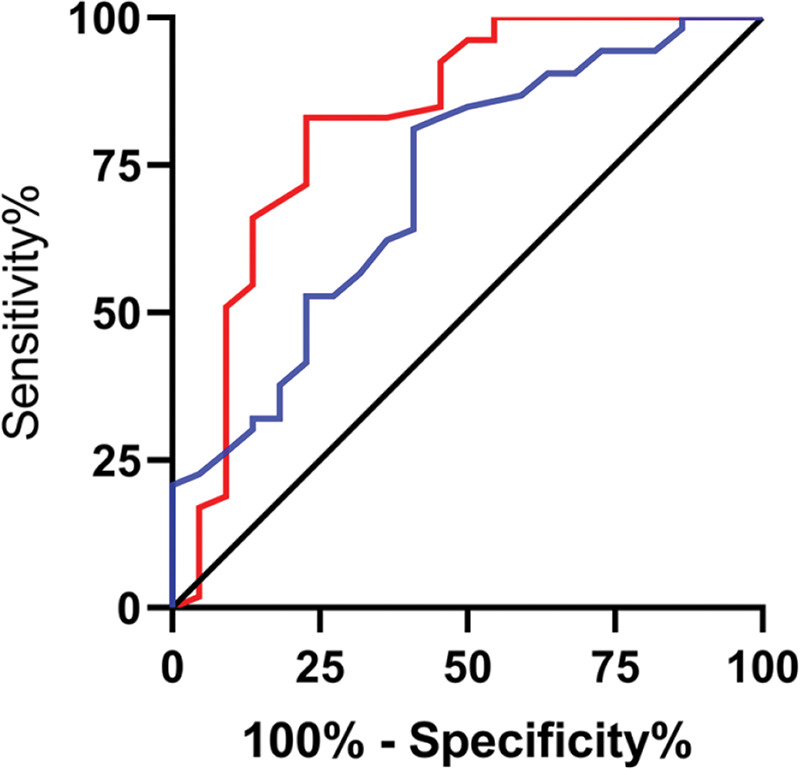
Predictive efficacy values of lncRNA H19 and *TET1* for UFs. A. The red line indicates the ROC curve of the predictive efficacy value of lncRNA H19 in UFs, with an AUC, sensitivity, specificity, and cut-off value of 0.814 (95% CI: 0.691-0.937), 81.13%, 77.27%, and <1.105, respectively. B. The blue line indicates the ROC curve of the predictive efficacy value of *TET1* in UFs, with an AUC, sensitivity, specificity, and cut-off value of 0.765 (95% CI: 0.647-0.884), 69.81%, 77.27%, and >0.915, respectively.

The expression levels of lncRNA H19 and *TET1* in the UF tissues before surgery were used to plot the ROC curves. The AUC of lncRNA H19 was 0.814 (95% CI: 0.691-0.937), with 81.13% sensitivity, 77.27% specificity, and a cut-off value of <1.105. The AUC of *TET1* was 0.765 (95% CI: 0.647-0.884), with 69.81% sensitivity, 77.27% specificity, and a cut-off value of >0.915 (Figure 5).

## DISCUSSION

Previous studies have shown that genetic factors, race, age, obesity, early menarche, caffeine intake, and alcohol intake are risk factors for UFs (23). UFs lead to infertility, heavy menstrual bleeding, recurrent abortion, and pelvic pain in women of childbearing age, with increased risk of adverse obstetric outcomes (e.g., cesarean section and postpartum hemorrhage) (24,25). Surgical resection is the preferred treatment for UFs, with laparoscopic resection being the gold standard method (26). However, Benaglia et al. reported that patients with UF have a recurrence rate of 21% after surgery (27). Therefore, it is of great significance to study the risk factors for postoperative recurrence of UFs and to identify valuable biological indicators for predicting recurrence.

Currently, UFs are mainly diagnosed using imaging in clinical practice (28). lncRNA H19 and *TET1* are considered important regulatory factors in the pathogenesis of UFs, but their diagnostic value remains unclear. In this study, we examined their expression levels in the serum of patients with UF and healthy controls. lncRNA H19 levels in the treated group were significantly higher than those in the control group, while *TET1* levels were significantly lower than those in the control group, indicating that they are potential diagnostic indicators of UFs. The AUCs of the ROC curves for lncRNA H19 and *TET1* were 0.872 and 0.826, respectively, suggesting that these two factors are valuable in diagnosing UFs. Recent studies have shown that lncRNA H19 is overexpressed in UFs and promotes cell proliferation (29). Lu et al. reported that miR-129 contributes to the occurrence of UFs by inhibiting *TET1* expression in UF tissues (30). We expect that the expression levels of lncRNA H19 and *TET1* mRNA after surgery often return to normal, making them valuable indicators of the efficacy of surgical treatment. However, whether the levels of lncRNA H19 and *TET1* mRNA in UF tissues before surgery can be used as predictors of efficacy remains unclear. To this end, we divided the patients into the BC (markedly effective) and PE groups (effective+ineffective). The results showed that compared with the PE group, lncRNA H19 expression level before surgery was significantly lower, while *TET1* expression level before surgery was significantly higher in the BC group. These results suggest that the expression levels of lncRNA H19 and *TET1* mRNA in UF tissues before surgery can be used as indicators for the short-term clinical efficacy of operation in patients. Therefore, these two factors will be useful for choosing the optimal treatment method for UFs, improving the clinical efficacy, and increasing the success rate of the treatment. Furthermore, we used the AUC of ROC curves to evaluate the predictive efficacy values of the two indicators and found that their AUCs were 0.788 and 0.812, respectively. Our results suggest that both lncRNA H19 and *TET1* have high predictive value and could be used to predict the efficacy of UF treatment.

The recurrence rate of UFs increases over time after both open surgery and laparoscopic resection, which often leads to additional surgery in patients (6). We followed up the 2-year postoperative recurrence of patients with UF and found that there were 22 patients (29.33%) with recurrence (DR group) and 53 patients (70.67%) with no recurrence (NR group). Univariate analysis of the clinical data showed that age >40 years, menarche age <13 years, maximum diameter of UFs >5 cm, number of UFs >4, high lncRNA H19 level, and low *TET1* level were risk factors for UF recurrence. The exact cause of postoperative recurrence of UFs remains unclear, but it is believed that small-sized UFs that are undetected during operation gradually grow after surgery due to various factors (31). In addition, pre-existing risk factors also contribute to the recurrence of UFs. Therefore, while surgery can remove existing UFs, surgical intervention that can reduce the risk of recurrence is still unavailable (32). This is due to the technical difficulty involved in the complete resection of multiple UFs, including tiny intramural residues. As a result, the small tumor foci continue to grow under the effect of various risk factors after the operation, eventually causing recurrence. Logistic regression analysis showed that age >40 years, menarche age <13 years, maximum diameter of UFs >5 cm, number of UFs >4, high lncRNA H19 level, and low *TET1* level were independent risk factors affecting UF recurrence. Hanafi et al. found that the 5-year postoperative recurrence rate of a single UF is significantly lower than that of multiple UFs (33), similar to our results. In our study, high lncRNA H19 levels and low *TET1* levels were independent risk factors for UF recurrence, suggesting that the levels of these two factors can be used as predictors of recurrence. Therefore, we examined lncRNA H19 and *TET1* levels in the UF tissues of patients in the DR and NR groups and analyzed the ROC curves. The results showed that the AUCs of lncRNA H19 and *TET1* were 0.814 and 0.765, respectively. This indicates that lncRNA H19 and *TET1* have high predictive values for postoperative recurrence of UFs.

In this study, we found that lncRNA H19 and *TET1* can be used as predictors for diagnosis, efficacy of operation, and postoperative recurrence of UFs. However, the role of lncRNAs and *TET1* in the pathogenesis of postoperative recurrence remains unclear. We also did not include data from postmenopausal women as a reference. In the future, we wish to further explore the mechanistic link between lncRNA H19, *TET1*, and UFs based on our current findings.

In summary, lncRNA H19 and *TET1* have high diagnostic and predictive efficacy values for UFs, and may potentially be used for predicting the postoperative recurrence of UFs.

## AUTHOR CONTRIBUTIONS

Zhan X, Zhou H and Chou C designed the experiments. Zhou H, Sun Y, and Shen B performed the experiments and analyzed the experimental results. Zhan X and Zhou H wrote the manuscript. Chou D revised the manuscript. All of the authors approved the final version of the manuscript.

## Figures and Tables

**Table 1:** Primer sequences.

Indicators	Upstream primers	Downstream primers
lncRNA H19	5′-TCCCAGAACCCACAACATGAA-3′	5′-TT-CACCTTCCAGAGCCGATTC-3′
*TET1*	5′-CTCCCATTCCTCCACCTTTG-3′	5′-CCACCACCCTGTTGCTGTAG-3′
*GAPDH*	5′-CCCACTCCTCCACCTTTGAC-3′	5′-GGATCTCGCTCCTGGAAGATG-3′

**Table 2:** Efficacy evaluation criteria for uterine fibroids (UFs).

Efficacy	Evaluation criteria
Markedly effective	The clinical symptoms were notably alleviated, and the UF volume reduced by ≥50%.
Effective	The clinical symptoms were partially alleviated, and the UF volume reduced by 10-49%.
Ineffective	The clinical symptoms were not alleviated or even worsened, and the UF volume either reduced by <10% or increased.

**Table 3:** Comparison of general information.

Factors		Treated group (n=75)	Control group (n=60)	t/χ2 value	*p*-value
Age (Years)		40.5±6.2	38.4±8.3	1.682	0.095
BMI (kg/m^2^)		21.32±1.78	21.64±1.81	1.030	0.305
History of smoking					
	Yes	26 (34.67)	19 (31.67)	0.616	0.433
	No	49 (65.33)	41 (68.33)		
History of alcoholism					
	Yes	34 (45.33)	32 (53.33)	0.854	0.356
	No	41 (54.67)	28 (46.67)		
Family history					
	Yes	16 (21.33)	15 (25.00)	0.253	0.615
	No	59 (78.67)	45 (75.00)		
Place of residence					
	City	43 (57.33)	27 (45.00)	1.521	0.217
	Countryside	32 (42.67)	33 (55.00)		
UFs location					
	Subserous	27 (36.00)			
	Intramural	23 (30.67)			
	Subserous and intramural	25 (33.33)			
Menarche age					
	<13 years old	26 (34.67)			
	≥13 years old	49 (65.33)			
Maximum diameter of UFs (cm)					
	<5	51 (68.00)			
	≥5	24 (32.00)			
Number of UFs					
	Single	42 (56.00)			
	Multiple	33 (44.00)			
Postoperative pregnancy					
	Yes	15 (20.00)			
	No	60 (80.00)			

Note: UFs, uterine fibroids.

Data conforming to a normal distribution are expressed as the mean±standard deviation (SD).

**Table 4:** Univariate analysis of postoperative recurrence.

Factors		DR group (n=22)	NR group (n=53)	t/χ2 value	*p-*value
Age (Years)					
	<40 (n=33)	16 (72.73)	17 (32.08)	10.43	0.001
	≥40 (n=42)	6 (27.27)	36 (67.92)		
BMI (kg/m^2^)		21.24±1.82	21.73±1.75	1.112	0.270
History of smoking					
	Yes (n=26)	11 (50.00)	15 (28.30)	3.232	0.072
	No (n=49)	11 (50.00)	38 (71.70)		
History of alcoholism					
	Yes (n=34)	13 (59.09)	21 (39.62)	2.378	0.123
	No (n=41)	9 (40.91)	32 (60.38)		
Family history					
	Yes (n=16)	6 (27.27)	10 (18.87)	0.654	0.419
	No (n=59)	16 (72.73)	43 (81.13)		
Place of residence					
	City (n=43)	12 (54.55)	31 (58.49)	0.099	0.753
	Countryside (n=32)	10 (45.45)	22 (41.51)		
UFs location					
	Subserous (n=27)	9 (40.91)	18 (33.96)	1.588	0.452
	Intermural (n=23)	8 (36.36)	15 (28.30)		
	Subserous and intramural (n=25)	5 (22.73)	20 (37.74)		
Menarche age					
	<13 years old (n=26)	15 (68.18)	11 (20.75)	11.54	<0.001
	≥13 years old (n=49)	7 (31.82)	42 (79.25)		
Maximum diameter of UFs (cm)					
	<5 (n=51)	6 (27.27)	45 (84.91)	18.73	<0.001
	≥5 (n=24)	16 (72.73)	8 (15.09)		
Number of UFs					
	Single (n=42)	5 (22.73)	37 (69.81)	13.99	<0.001
	Multiple (n=33)	17 (77.27)	16 (30.19)		
Postoperative pregnancy					
	Yes (n=15)	6 (27.27)	9 (16.98)	1.029	0.310
	No (n=60)	16 (72.73)	44 (83.02)		
lncRNA H19 (median)					
	<1.120	7 (31.82)	45 (84.91)	20.61	<0.001
	≥1.120	15 (68.18)	8 (15.09)		
*TET1* (median)					
	<0.902	16 (72.73)	14 (26.42)	13.89	<0.001
	≥0.902	6 (27.27)	39 (73.58)		

Note: UFs, uterine fibroids. Data conforming to a normal distribution are expressed as the mean±SD.

**Table 5:** Assignment of factors showing marked differences.

Factors	Assignment
Recurrence (Y)	Recurrence=1, no recurrence=0
Age	<40=1, ≥40=0
Menarche age	<13=1, ≥13=0
Maximum diameter of UFs	≥5=1, <5=0
Number of UFs	Multiple=1, single=0
lncRNA H19 level	≥1.120=1, <1.120=0
*TET1* level	<0.902=1, ≥0.902=0

Note: UFs, uterine fibroids.

**Table 6:** Multivariate logistic regression.

Factors	B	S.E.	χ2	*p*-value	Exp (B)	95% C.I. for EXP (B)	
						Lower	Upper
Age	1.077	0.524	4.223	0.040	2.936	1.051	8.201
Menarche age	1.094	0.465	5.537	0.019	2.986	1.201	7.428
Maximum diameter of UFs	1.000	0.391	6.529	0.011	2.719	1.262	5.856
Number of UFs	0.972	0.387	6.319	0.012	2.644	1.239	5.643
lncRNA H19 level	0.985	0.396	6.542	0.010	2.741	1.270	5.873
*TET1* level	0.934	0.356	6.127	0.014	2.462	1.194	5.387

Note: UFs, uterine fibroids; S.E., standard error.

Data conforming to a normal distribution are expressed as the means.
